# Characterization of the interleukin-17 effect on articular cartilage in a translational model: an explorative study

**DOI:** 10.1186/s41927-020-00122-x

**Published:** 2020-05-12

**Authors:** Dovile Sinkeviciute, Anders Aspberg, Yi He, Anne-Christine Bay-Jensen, Patrik Önnerfjord

**Affiliations:** 1 Nordic Bioscience, Biomarkers & Research, Herlev, Denmark; 2 Rheumatology and Molecular Skeletal Biology, Department of Clinical Sciences Lund, Lund University, Lund, Sweden

**Keywords:** Osteoarthritis, Inflammation, Interleukin-17

## Abstract

**Background:**

Osteoarthritis (OA) is a progressive, chronic disease characterized by articular cartilage destruction. The pro-inflammatory cytokine IL-17 levels have been reported elevated in serum and synovial fluid of OA patients and correlated with increased cartilage defects and bone remodeling. The aim of this study was to characterize an IL-17-mediated articular cartilage degradation ex-vivo model and to investigate IL-17 effect on cartilage extracellular matrix protein turnover.

**Methods:**

Full-depth bovine femoral condyle articular cartilage explants were cultured in serum-free medium for three weeks in the absence, or presence of cytokines: IL-17A (100 ng/ml or 25 ng/ml), or 10 ng OSM combined with 20 ng/ml TNFα (O + T). RNA isolation and PCR analysis were performed on tissue lysates to confirm IL-17 receptor expression. GAG and ECM-turnover biomarker release into conditioned media was assessed with dimethyl methylene blue and ELISA assays, respectively. Gelatin zymography was used for matrix metalloproteinase (MMP) 2 and MMP9 activity assessment in conditioned media, and shotgun LC-MS/MS for identification and label-free quantification of proteins and protein fragments in conditioned media. Western blotting was used to validate MS results.

**Results:**

IL-17RA mRNA was expressed in bovine full-depth articular cartilage and the treatment with IL-17A did not interfere with metabolic activity of the model. IL-17A induced cartilage breakdown; conditioned media GAG levels were 3.6-fold-elevated compared to untreated. IL-17A [100 ng/ml] induced ADAMTS-mediated aggrecan degradation fragment release (14-fold increase compared to untreated) and MMP-mediated type II collagen fragment release (6-fold-change compared to untreated). MS data analysis revealed 16 differentially expressed proteins in IL-17A conditioned media compared to untreated, and CHI3L1 upregulation in conditioned media in response to IL-17 was confirmed by Western blotting.

**Conclusions:**

We showed that IL-17A has cartilage modulating potential. It induces collagen and aggrecan degradation indicating an upregulation of MMPs. This was confirmed by zymography and mass spectrometry data. We also showed that the expression of other cytokines is induced by IL-17A, which provide further insight to the pathways that are active in response to IL-17A. This exploratory study confirms that IL-17A may play a role in cartilage pathology and that the applied model may be a good tool to further investigate it.

## Background

Articular cartilage – an aneural, avascular and alymphatic connective tissue that can withstand compressive load – provides a low friction gliding surface, facilitating synovial joint articulation [1, 2]. It is composed of chondrocytes (1–5% of the cartilage tissue volume) and a dense extracellular matrix (ECM), which primarily consists of water (up to 80% of wet cartilage weight), proteoglycans and collagens [1]. The ECM mainly provides the biomechanical properties and function of cartilage while chondrocytes regulate cartilage homeostasis and, thus, are key for its development, maintenance and repair [1, 2].

Osteoarthritis (OA) is a degenerative joint disease, characterized by progressive articular cartilage destruction, subchondral bone remodeling and occasional synovial inflammation [3]. It is the most common joint disease worldwide and one of the most frequent health problems experienced by middle aged and older people [3, 4]. It affects women more frequently than men, is more common in the developed world and has a negative impact on patient well-being [3, 5, 6]. Obesity, gender, age, genetics, diet, joint injury, abnormal joint loading and joint malalignment are risk factors for OA [3, 7].

OA is a heterogenous disease thought to be composed of multiple phenotypes, each with a different underlying driver, e.g.: age, cartilage damage, metabolic dysregulation, subchondral bone remodeling, and inflammation [4]. In a healthy joint a fine balance of cartilage synthesis and degradation is maintained, but in OA cartilage the homeostasis is disrupted by mechanisms driven by a combination of biological mediators and biomechanical stimuli [2]. This leads to over-active inflammatory, degradation and synthesis processes, which are mediated by cytokines, matrix-degrading proteinases and growth factors [2]. Inflammatory cytokines are among the most important and best documented factors participating in the pathogenesis of joint disease and cartilage degradation [8]. They contribute to the destruction of articular cartilage via 1) decreasing the synthesis of key ECM components, 2) increasing the release of proteolytic enzymes that degrade articular cartilage, such as various MMP (matrix metalloproteinase) and ADAMTS (A Disintegrin and Metalloproteinase with Thrombospondin motifs) enzymes, and 3) release of inflammatory PGE_2_ (prostaglandins), COX-2 (cyclooxygenase 2), nitric oxide (NO) and free radicals into the intraarticular space [2, 9]. It is now generally accepted that tumor necrosis factor alpha (TNFα) is one of the key cytokines in the process of chronic joint inflammation and subsequent changes in cartilage and bone; its serum and synovial fluid levels correlate positively with disease scores in both OA and RA patients [8]. Anti-TNF therapy in RA is already FDA and EMA approved and reportedly shows chondroprotective effects [8]. Furthermore, TNFα can synergize with other cytokines, such as interleukin-6 (IL-6), interleukin-17 (IL-17), or oncostatin M (OSM) to further promote matrix turnover and cartilage destruction [10–13].

The IL-17 pro-inflammatory cytokine family has only more recently gained attention in the OA field [14]. The family consists of six ligands: IL-17A, IL-17B, IL-17C, IL-17D, IL-17E (also known as IL-25), and IL-17F, and five corresponding receptors (IL-17RA, IL-17RB, IL-17RC, IL-17RD, IL-17RE) [15]. IL-17A is the most extensively studied of the family and is often referred to as simply “IL-17”. IL-17A signals through a IL-17RA/IL-17RC heterodimeric receptor complex as a homodimer or a heterodimer with IL-17F; the receptor activation leads to recruitment of intermediate adaptor protein Act1, and activation of several signaling pathways: the canonical nuclear factor kappa light chain enhancer of activated B cells (NFκB) pathway, the mitogen-activated protein kinase (MAPK) signaling pathway, as well as C/EBPs, JAK-PI3K and JAK-STAT pathways [16–19]. As a result, IL-17 mediates angiogenesis, recruitment of inflammatory cells, and induction of proinflammatory mediator release [16]. IL-17 receptor is expressed in vivo in chondrocytes from different arthropathies (RA, OA, and seronegative spondyloarthropathies) [20], and IL-17 is implicated in multiple joint pathologies, such as psoriatic arthritis, ankylosing spondylitis and rheumatoid arthritis [21, 22], and drugs targeting IL-17 have been approved for treatment of psoriatic arthritis [23]. In OA patients, elevated serum and synovial fluid levels of IL-17 were reported to show a positive correlation with radiographic features of the disease [24–29]. The physiological levels in healthy and OA subjects in these studies were in the order of 1–10 pg/ml in both serum and synovial fluid. Furthermore, in certain populations polymorphisms in the IL-17A gene may correlate with susceptibility to OA [30].

There are currently no clinically approved disease-modifying drug (DMOAD) treatments for OA. The clinical trial failure rate for new drugs is relatively high, due to, in part, the heterogeneity of the OA patient population [4, 31], thus a better understanding of articular cartilage biology, particularly in relation to its degradation would facilitate a better subtyping of patients and benefit the screening of novel drug candidates.

Kjelgaard-Petersen et al. (2018) have previously shown that full-depth bovine articular cartilage explants (BEX) treated with oncostatin M in combination with TNFα (O + T) were a reliable translational model for rheumatoid arthritis by successfully back-translating the serological biomarker results from a phase III clinical study (OSKIRA-1) [32]. Proteomic technologies allow for a large-scale unbiased investigation, making it possible to identify factors involved in joint disease progression and build a library of mediators: previous studies have revealed numerous cytokines, proteases, and matrix fragments in serum, synovial fluid, and articular cartilage of OA patients [33–36]. Therefore, adapting the BEX model for studying IL-17-mediated articular cartilage changes and combining it with proteomics could contribute to a better understanding of molecular events involved in IL-17-mediated cartilage turnover and identification of its biomarkers. The ability to better identify IL-17-mediated processes in cartilage would benefit both clinical studies and the clinic, through better patient selection and better matching of the available anti-IL-17 therapies to the patients, respectively. In this pilot study we aim to characterize a model of IL-17 mediated knee cartilage destruction and to investigate IL-17 effects on articular cartilage protein turnover by using biomarker assays and proteomics for analysis of proteins released into explant media, while benchmarking the effect to O + T.

## Methods

### Materials and buffers

Bovine knees were obtained from the local abattoir (Harald Hansens Eftf. I/S, Denmark). Biopsy punchers were purchased from Scandidact (cat#MTP-33-32). Dulbecco’s Modified Eagle medium (DMEM)/F12-GlutaMAX™ purchased from Thermo Fisher (cat#31331–093), together with Penicillin and Streptomycin (P/S) (cat#4333). The cytokines OSM (cat#O9635, Sigma-Aldrich), TNFα (R&D Systems, cat#210-TA), IL-17A (R&D Systems, cat#7955-IL) were reconstituted in 0.1% bovine serum albumin (BSA) and further diluted in culture medium. All chemicals were purchased from Sigma-Aldrich or Merck unless stated otherwise.

### Full-depth bovine articular cartilage explant tissue culture

Intact healthy stifle joints (i.e. with no perforation of the synovial capsule) from < 2-year-old cattle were used within 48 h after slaughter. After dissection and exposing the articular cartilage under sterile conditions, full-depth, 3 mm-in-diameter bovine cartilage explants (BEX) were punched out from the medial and lateral condyle and released from the subchondral bone with a sharp scalpel. One explant per well were placed in 96-well plates and washed five times in explant culture medium (DMEM+Glutamax/F12 (1:1) + 1% Penicillin + 1% Streptomycin). Plates were pre-incubated overnight at 37 °C, 5% CO_2_, ambient oxygen level for explants to equilibrate. Next day, explant metabolic activity was assessed using the alamarBlue (resazurin) metabolic assay [37] and fresh media containing treatments (10 ng/ml OSM in combination with 20 ng/ml TNFα; 100 ng/ml IL-17A; 25 ng/ml IL-17A or media without any treatment were added to an equal number of explants. Previous explant studies have used IL-17 levels in the range of 0.4–100 ng/ml [38–40]. Based on preliminary cartilage explant experiments assessing collagenolytic activity in response to IL-17A (Supplementary figure 1), we selected our upper level to be 100 ng/ml with the additional dose of 25 ng/ml for comparison in our model. Both these concentrations are higher than reported physiological levels in serum and synovial fluid. However, in order to keep the experiments within a reasonable time frame this compromise was made in this exploratory study. Explants, with or without stimuli, were cultured at 37 °C, 5% CO_2_, ambient oxygen level for 21 days with media changes and conditioned media harvests performed three times a week, and metabolic activity assessed every seven days. Conditioned media from two animals (six technical replicates – explants – per condition, per animal) were selected for biomarker analysis. For proteomics analysis, conditioned media from additional two animals (two technical replicates – explants – per condition, per animal) were used.

### Explant metabolic activity assessment using alamarBlue assay

Two hundred μl of 10% alamarBlue (Thermo Fisher Scientific, cat#DAL1100) in BEX culture media solution was added to each well containing an explant and 4 empty wells for background. The 96-well plates were incubated for 3 h at 37 °C, 5% CO_2_, after which 160 μl of alamar media was transferred to a black 96-well plate and read in a fluorimeter (Ex: 540 nm, Em: 590 nm, SpectraMax M5, Molecular Devices). The metabolic activity was measured in a total of 24 explants per each treatment group: 6 explants for each treatment group per animal; tissue from 4 animals.

### PCR for IL-17R expression verification

Healthy, full-depth bovine femoral condyle articular cartilage (2.5 g) from two individual animals was snap-frozen and pulverized using a mortar and pestle in liquid nitrogen. Total RNA was extracted using RNAqueous Micro Kit (Thermo Fisher Scientific, cat#AM1912) according to the manufacturer’s instructions. Briefly, 4 ml of Lysis/Binding solution was added to 400 mg of pulverized cartilage and the lysate was homogenized using a rotor-stator homogenizer and centrifuged to 3 min at 16,000 x g to remove debris. The supernatant was mixed thoroughly with an equal volume of 64% ethanol and loaded onto Filter Cartridges (700 μl per cartridge). After washing, RNA from each cartridge was eluted using 40 μl + 10 μl of the kit’s elution solution. The eluates were combined, and the RNA amount was spectrophotometrically determined (Nanodrop2000, Thermo Fisher Scientific). cDNA synthesis was performed with the Maxima First Strand cDNA Synthesis Kit for RT-qPCR (Thermo Fisher Scientific, cat#K1641), using 50 ng total RNA in a total volume of 20 μl and incubated at: 25 °C (10 min), 50 °C (15 min) and 85 °C (5 min). IL-17 receptor amplification was performed using Dream Taq Green PCR kit, with 0.5 ng (assuming 1:1 conversion from RNA) template cDNA and 200 nM (each) primers for IL-17RA (bIL17RA_f1: GCCCATTCCCAGGAGCTAAA, bIL-17RA_r1: CCACTGACCAAGACCACACA, product size: 459 bp), IL-17RB (bIL17RB_f1: ATCACCTTGGCTGCACATCA, bIL-17RB_r1: AAAACGTCCATTTGCCACCA, product size: 444 bp), IL-17RC (bIL17RC_f2: TCTCTGGGATGGTGACGTGC, bIL-17RC_r2: AGGATCTTCCCAGTACCCCT, product size: 178 bp), IL-17RD (bIL17RD_f1: TAAAACCAGTGCATTCCCCGT, bIL-17RD_r1: ACCCATTCTCTCTGCCGTTC, product size: 364 or 433 bp), or IL-17RE (bIL17RE_f1: GGAGCCACACTGTAGACCTG, bIL-17RE_r1: ACATACCACCCCTCTGACTCT, product size: 307 bp). The amplification was performed in a SimpliAmp PCR apparatus (Applied Biosystems) and the cycle conditions were: 95 °C (3 min), 40 cycles of 95 °C (30 s), 55 °C (30 s), 72 °C (1 min), followed by 72 °C (10 min). The PCR products were separated on a 2% agarose gel (containing ethidium bromide) by electrophoresis and visualized by UV light in a ChemiDoc MP System (BioRad). PCR product bands were detected, and size estimated by ImageLab 6.0.1 software (BioRad). The gel image was exported as a TIFF file, cropped and annotated using Affinity Design 1.7.

### Sulfated glycosaminoglycan (GAG) release measurement using a DMMB assay

The 1,9-dimethylmethylene blue (DMMB, Sigma-Aldrich, cat#341088) assay with chondroitin sulfate (Sigma, cat#C9819) as standard was used to measure the concentration of released GAGs from the cartilage explants in the culture medium [41, 42]. The GAG content was determined by adding 250 μl DMMB solution (0.05 mM DMMB, 41 mM NaCl, 45 mM glycine and pH = 3.0) to 20 μl supernatant sample in a 96-well plate and immediately measuring the colorimetric change using a plate reader (SpectraMax M5, Molecular Devices) at 570 nm.

### Histological analysis

To visualize proteoglycans in the tissue, cartilage explants were fixed on the last day of culture using 4% phosphate-buffered formalin (pH 7.0), dehydrated with an automated tissue-processing apparatus (Sakura Tissue Tek VIP 5) and embedded in paraffin. The embedded tissue was cut to 5-μm thickness with the microtome knife angled at 9–10 degrees. The sections were collected on Superfrost+ glass slides (Thermo Scientific, cat# J1800AMNT), stained with safranin O/fast green (Brunschwig Chemie) and mounted with Eukitt mounting medium (Sigma-Aldrich, cat# 03989). The slides were analyzed on a Zeiss Axioplan-2 imaging system (Carl Zeiss). The samples were viewed through a 10x objective and images captured with an AxioCam HR camera using AxioVision 4.2 (Carl Zeiss) software.

### Gelatin zymography

Pre-cast Novex™ Zymogram Plus Gels (Thermo Fisher Scientific, cat#ZY00100BOX), 10X Tris-Glycine SDS Running Buffer (Thermo Fisher Scientific, cat#LC2675), 10X Novex™ Zymogram Renaturing Buffer (Thermo Fisher Scientific, cat#LC2670) and 10X Novex™ Zymogram Developing Buffer (Thermo Fisher Scientific, cat#LC2671) and the XCell SureLock Mini-Cell (Thermo Fisher Scientific) gel running system was used for gelatin zymography, following the manufacturer’s instructions. Briefly, 15 μl of sample was diluted 1:1 in 2X Tris-Glycine SDS Sample Buffer. 20 μl of samples were loaded onto the 1.0 mm, 10-well gelatin gel, immersed in 1X Tris-Glycine SDS Running Buffer and run for 105 min at constant 125 V. The gel was renatured and equilibrated as recommended and developed in 1X Zymogram Developing Buffer at 37 °C for 24 h. The gel was stained with SimplyBlue Safestain (Thermo Fisher Scientific, cat#LC6060) using the high-sensitivity protocol.

### Western blotting

Pooled conditioned media (from days 10, 14, 19 and 21) was diluted in NuPAGE 4X LDS Sample Buffer (Thermo Fisher Scientific, cat#NP0007), heated at 70 °C for 10 min, then 20 μl was loaded onto a pre-cast NuPAGE 4–12% Bis-Tris 1.0 mm mini gel (Thermo Fisher Scientific, cat#NP0321BOX). The gel was run at 50 V for 30 min followed by 70 V for 1.5 h in 1X MES SDS buffer (Thermo Fisher Scientific, cat#NP0002). The protein transfer to nitrocellulose membrane was performed using P3 program on iBlot 2 Dry Blotting System (Thermo Fisher Scientific) according to manufacturer’s instructions. After blocking non-specific binding for 1 h at room temperature with 5% BSA in Tris-buffered saline, 0.1% Tween 20 (TBST), the membrane was incubated with primary antibody against CHI3L1 (1:500, rabbit anti-human CHI3L1, Abcam, cat#ab180569) in 5% BSA TBST. After washing, the membrane was incubated with secondary antibody (1:50000, Jackson ImmunoResearch, cat#111–035-003) in 5% BSA TBST for 1 h at room temperature. Signals were visualized with SuperSignal West Femto chemiluminescence kit (Thermo Fisher Scientific, cat#34094) on ChemiDoc MP System (BioRad). The blot image was exported as a TIFF file, cropped and annotated using Affinity Design 1.7.

### Cartilage degradation biomarker measurements

Cartilage degradation was measured using three protein neo-epitope biomarker enzyme-linked immunosorbent assays (ELISAs): C2M (Nordic Bioscience, cat#1100), C3M (Nordic Bioscience, cat#1200) and exAGNx1 (Nordic Bioscience, in-house kit), using a method described previously [43–45]. Briefly, 96-well streptavidin-coated microtiter plates were coated for 30 min at 20 °C with the respective biotin-labelled neo-epitope peptides. Unbound peptide was washed off and 20 μL samples/standards were added, followed by 100 μl/well of peroxidase-labeled (C2M, C3M) or unlabeled (exAGNx1) monoclonal antibody in an assay-specific buffer. The plates were incubated either overnight at 4 °C (C2M), for 3 h (exAGNx1) at 20 °C, or for 1 h (C3M) at 20 °C. ExAGNx1 was additionally incubated for 1 h with a secondary anti-mouse horseradish peroxidase (HRP) labelled antibody after the 3-h incubation with the primary antibody. The plates were washed and developed using 3,3′,5,5′-Tetramethylbenzidine (TMB) and the reaction was stopped with sulfuric acid after 15 min. All assays were read on a standard plate reader (SpectraMax M5, Molecular Devices) at 450 nm with reference at 650 nm and calibration curves were plotted using a four-parametric mathematical fit model.

### Sample processing and mass spectrometry for analysis of protein release from articular cartilage

Fifty μl of conditioned culture medium from different time points (days 10, 14, 19 and 21 of culture) was reduced by 4 mM dithiothreitol for 30 min at 56 °C, alkylated by 16 mM iodoacetamide for 60 min in the dark at room temperature, precipitated with ethanol and digested by 0.25 μg trypsin gold (Promega, cat#V5280) in 100 μl 0.1 M ammonium bicarbonate (AMBIC) buffer pH 7.8 for 16 h on a shaker at 37 °C. After addition of 100 μl 1 M NaCl with 1% formic acid to the digests, these were run through 30 kDa filters (PALL Life Sciences, cat#OD030C34) to remove GAGs and desalted with reversed-phase Vydac UltraMicro Spin C18 columns (Harvard Apparatus, cat#74–7206) according to the manufacturer’s instructions. Non-targeted mass spectrometry analysis was performed on a quadrupole Orbitrap benchtop mass spectrometer, QExactive, (Thermo Scientific) equipped with an Easy nano-LC 1000 system (ThermoFisher Scientific). Separation was performed on 75 μm × 25 cm, Acclaim Pepmap™ RSLC C18 capillary columns packed with 2 μm particles (ThermoFisher Scientific). A spray voltage of + 2000 V was used with a heated ion transfer setting of 275 °C for desolvation. The on-line reversed-phase separation was performed using a flow rate of 300 nl/min and a linear binary gradient 85 min was used. The gradient started with 3% solvent B for 4 min, then going to 35% solvent B in 64 min, after which it goes to 45% solvent B in 5 min. Finally, the organic solvent concentration was increased up to 90% in 5 min and kept at 90% for 7 min. An MS scan (400–1200 m/z) was recorded in the Orbitrap mass analyzer set at a resolution of 70,000 at 200 m/z, 1 × 10^6^ automatic gain control (AGC) target and 100 ms maximum ion injection time [46]. The MS was followed by data-dependent collision-induced dissociation MS/MS scans at a resolution of 17,500 on the 15 most intense multiply charged ions at 2 × 10^4^ intensity threshold, 2 m/z isolation width and dynamic exclusion enabled for 30 s.

### MS data analysis

Identification from discovery data was performed using *the Bos taurus* proteome (UniProt proteome ID UP000009136, n23868, downloaded 08/06/2015) with Proteome Discoverer 2.3 software (ThermoFisher Scientific). The processing workflow consisted of the following nodes: Spectrum Selector for spectra pre-processing (precursor mass range: 350–5000 Da; S/N Threshold: 1.5), Sequest-HT search engine (Protein Database: see above; Enzyme: Trypsin; Max. missed cleavage sites: 2; Peptide length range 6–144 amino acids; Precursor mass tolerance: 10 ppm; Fragment mass tolerance: 0.02 Da; Static modification: cysteine carbamidomethylation; and Percolator for peptide validation (FDR < 1% based on peptide q-value). Results were filtered to keep only the Master protein with at least one unique peptide, and protein grouping was allowed according to the parsimony principle. For label-free quantification (LFQ), the sum of the top 3 peptides for each protein was taken to reflect the intensity of the protein. Peptide intensities were quantified using a proprietary algorithm developed in Proteome Discoverer 2.3 (ThermoFisher Scientific).

### Statistical analysis and data visualization

Biomarker measurements below Lower Limit of Measurement Range (LLMR) were imputed as the LLMR of the individual biomarker. Biomarker measurements above Upper Limit of Measurement Range (ULMR) were imputed as the ULMR of the individual biomarker and not re-measured due low remaining sample volume. The release of GAGs and biomarkers over time was quantified by plotting the concentration in the medium against time in culture and calculating the area under the curve (AUC) using GraphPad Prism 7 for each BEX explant. For biomarker AUCs the baseline was set at the LLMR for the individual biomarker. The distribution of the AUC values was tested using R (version 3.4.0) by plotting histograms and quantile-quantile (Q-Q) plots. Since the GAG and biomarker data did not follow a Gaussian distribution a non-parametric Kruskal-Wallis with Dunn’s multiple comparison test was used to test for differences of the AUCs of GAG, AGNx1, C2M and C3M between the treatments, where *p* < 0.05 was considered significant.

The proteomics data was exported from Proteome Discoverer for statistical data analysis and visualization using R (version 3.5.1). The individual explant (technical replicate) LFQ values were log2-transformed and averaged, giving mean log2 value per treatment per timepoint for each animal. The data was then filtered so that only proteins present in both animals in both the untreated and the IL-17-treated samples were retained. Then, AUC was calculated to obtain a single value per the treatment group (untreated or IL-17A) within each animal, and the differences between untreated and IL-17 groups were compared for each protein using a reproducibility-optimized test statistic (ROTS) from ROTS package [47–50] (B = 1000), with adjusted *p*-value of < 0.05 and FDR of < 0.05 considered significant .

## Results

### IL-17 receptor mRNA is expressed in bovine full-depth articular cartilage

**Fig. 1:**
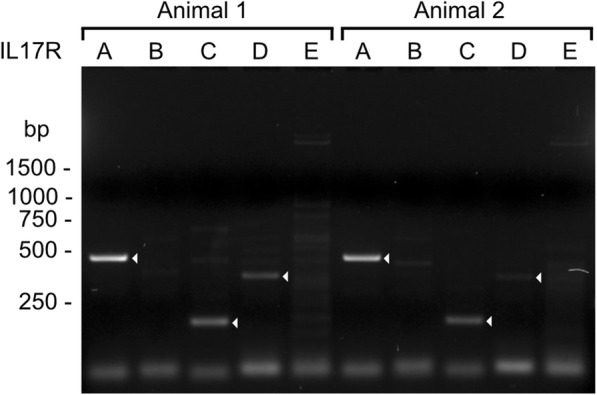
IL-17 receptor expression analysis in bovine articular cartilage by PCR and agarose gel (2%) electrophoresis. mRNA was extracted from the cartilage tissue of two animals, obtained within 48 h after slaughter, reverse-transcribed to cDNA, and 0.5 ng of template cDNA was amplified with IL-17 receptor primers. In both animals, PCR products for IL-17RA (lanes labeled A, expected size 459 bp) and IL-17RC (lanes C, expected size 178 bp) were observed. Additionally, PCR products for IL-17RD (lanes D, expected 364 or 433 bp) were also observed, albeit very weak in animal 2. Lanes are labeled with letters denoting the IL17R variant amplified and bands corresponding to the expected product sizes are indicated with white arrowheads

In order to confirm that our model system can respond to IL-17 stimulus, IL-17A receptor expression was verified in freshly harvested full-depth bovine stifle joint articular cartilage tissue biopsies by PCR and 2% agarose gel electrophoresis. An approximately 445 bp PCR product corresponding to IL-17RA (calculated size: 459 bp) – the receptor for IL-17A – was observed in both animals analyzed (Fig. 1). Additionally, weaker PCR products were observed at approximately 185 bp for IL-17RC (calculated size: 178 bp) and at approximately 350 bp for IL-17RD (calculated size: 364 or 433 bp, dependent on splicing). Thus, at least IL-17R A and C are expressed in bovine articular cartilage, while IL-17RD expression appears variable.

### Treatment with IL-17A does not interfere with the metabolic activity of the model

**Fig. 2:**
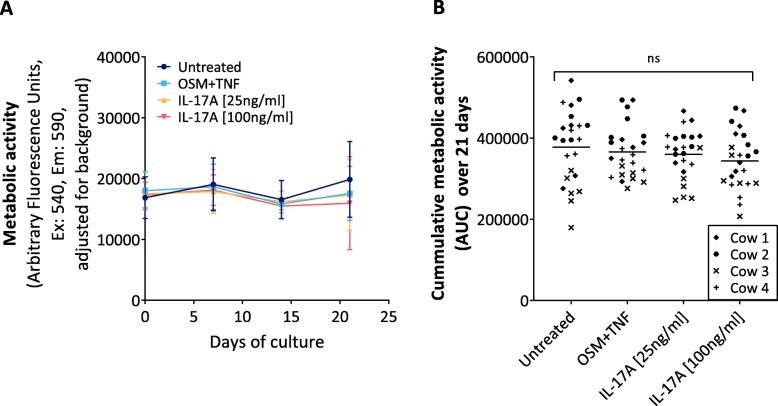
AlamarBlue® metabolic activity assessment of the bovine articular cartilage explants over a three-week culture period. **a** Metabolic activity, reported as a fluorescence (590–540 nm) readout, at day 0, 7, 14 and 21 in conditioned media from untreated BEX explants, or BEX explants treated with OSM [10 ng/mL] + TNFα [20 ng/mL], or IL-17A [100 ng/ml or 25 ng/ml]. **b** Area under the curve (AUC) was calculated from each explant’s fluorescence readout over time. Data is presented as means; bars represent SD and ns indicates no statistically significant difference between either one of the treatment groups compared to the untreated group (Kruskall-Wallis test with Dunn’s post-hoc test for multiple comparisons where *p* < 0.05 was considered significant)

Next, to verify that our explant system remains viable throughout the three-week tissue culture period and, thus, that any observed changes to protein release into conditioned media are not due to tissue death, the metabolic activity of explants was assessed every 7th day of the culture period using a commercial resazurin-based metabolic assay. There was no statistically significant reduction in metabolic activity over three weeks tissue culture period between the cytokine-treated explants and the untreated explants (Fig. 2a and b).

### IL-17A induces extracellular matrix breakdown in the explant model

**Fig. 3:**
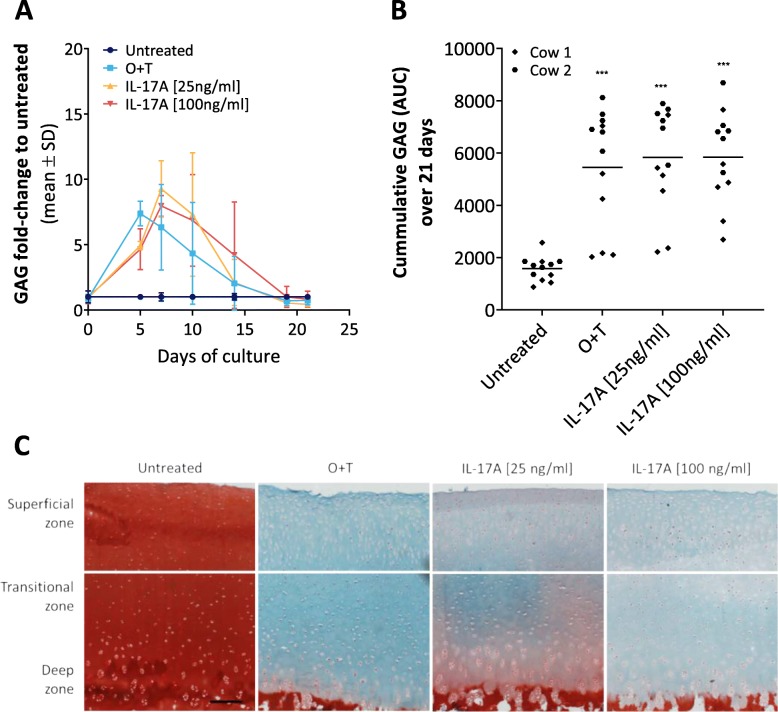
Glycosaminoglycan content in conditioned media and explant tissue. **a** GAG release into conditioned media over a 3-week culture period as measured by a DMMB assay; data is represented as a fold-change to untreated. **b** Cumulative release of GAG into the medium over the entire culture period, determined as the AUC calculated from each explant’s GAG release over time. Asterisks (*) represent statistical significance level compared to untreated: ***P < 0.001. **c** Safranin O/Fast green staining of bovine full-depth articular cartilage explants on the final day of tissue culture in the presence or absence of pro-inflammatory cytokines (magnification: 10×, scale bar: 200 μm). Note the pronounced loss of proteoglycan (red Safranin O staining) in cytokine-treated explants compared to the untreated explants

The ability of IL-17 to induce degradation of glycosaminoglycans has been previously described in other ex-vivo models [39, 40]. In order to verify this in our model, sulfated GAG release into conditioned media was measured over the three week-long culture period by a DMMB colorimetric assay and visualized by safranin O/fast green F histological staining of explant tissue section. IL-17A at both lower (25 ng/ml) and higher (100 ng/ml) concentrations induced GAG release from bovine knee full-depth articular cartilage explants in a time-dependent manner, as did O + T, which was used in the model as a positive control of cartilage catabolic responsiveness (Fig. 3a). Compared to the untreated group the overall conditioned media GAG levels were, on average, 3.67-fold higher in the IL-17A[100 ng/ml] group, 3.65-fold higher in in the IL-17A[25 ng/ml group] and 3.4-fold higher in the O + T group (Fig. 3b). In the explant tissue, in contrast, very little proteoglycan (safranin O) staining remained in any of the three cytokine-treated explant groups at day 21, whereas a normal cartilage structure was preserved in untreated explants (Fig. 3c-d).

**Fig. 4:**
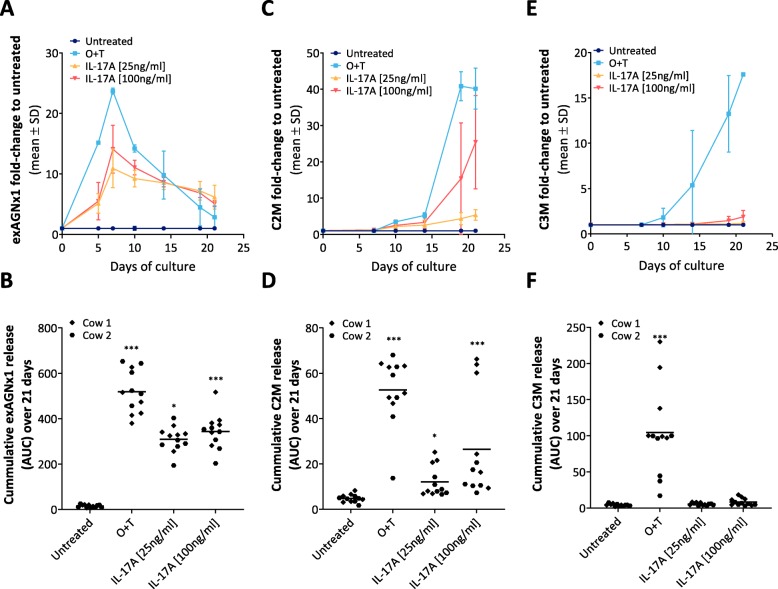
Cartilage ECM turnover serological biomarker release into explant conditioned media. The full-depth bovine articular cartilage explants were cultured either in absence (untreated), or in the presence of a catabolic stimuli: OSM [10 ng/ml] + TNFα [20 ng/ml], or IL-17A [100 ng/ml, 25 ng/ml] for 21 days with conditioned media exchanged three times a week. **a** ADAMTS-mediated aggrecan degradation fragment exAGNx1 release into conditioned media measured at day 0, 5, 7, 10, 14, 19 and 21 represented as fold-change to untreated. **b** AUC, as an indication of total signal, was calculated from each explant’s AGNx1 release over time. **c** MMP-mediated type II collagen degradation fragment C2M release into conditioned media measured at day 0, 7, 10, 14, 19 and 21 represented as fold-change to untreated. **d** AUC, as an indication of total signal, was calculated from each explant’s C2M release over time. **e** MMP-mediated type III collagen degradation fragment C3M release into conditioned media measured at day 0, 7, 10, 14, 19 and 21 represented as fold-change to untreated. **d** AUC, as an indication of total signal, was calculated from each explant’s C3M release over time. Asterisks (*) represent statistical significance level compared to untreated: *P < 0.05; ***P < 0.001

To further investigate the effect of IL-17A on ECM turnover of explants, and to confirm the usefulness of serological ECM turnover biomarkers in our model, degradation fragments of major cartilage structural proteins were measured by competitive sandwich ELISA. The biomarker exAGNx1 (NITEGE^373^ fragment from ADAMTS-mediated aggrecan degradation was measured on days 0, 5, 7, 10, 14, 19 and 21 of culture. The exAGNx1 levels increased in the cytokine-treated explant conditioned media, peaking at day 7: 24-fold for O + T group, 11-fold for IL-17A[25 ng/ml] and 14-fold for IL-17A[100 ng/ml] group when compared to the untreated group (Fig. 4a). ExAGNx1 levels then decreased over the next two weeks but did not reach the levels of untreated group by the end of the tissue culture period (Fig. 4a). Overall, the level of exAGNx1, calculated as area under the curve (AUC), was statistically significantly higher in both IL-17A groups and the positive control (O + T) group when compared to the untreated group (Fig. 4b).

As the collagen fibrillar network is a major component of the ECM, we also investigated the IL-17A effects on articular cartilage collagens by measuring biomarkers for MMP-mediated type II collagen (C2M) and type III collagen (C3M) degradation. The biomarkers were assessed in the conditioned media at days 0, 7, 10, 14, 19 and 21. Like with aggrecan degradation, the release of C2M fragment into the conditioned media increased over time in the cytokine-treated groups (Fig. 4c) and the overall amount of the biomarker in the conditioned media was, on average, 6-fold higher in IL-17A[100 ng/ml] explants, 3-fold higher in IL-17A[25 ng/ml] explants, and 13-fold higher in O + T explants when compared to the untreated group, which was statistically significant (Fig. 4d). Although C3M release into conditioned media also increased over time in the positive catabolic control O + T group and there was some induction observed in the IL-17[100 ng/ml] group (1.9-fold increase at day 21) (Fig. 4e), overall, there was no statistically significant C3M increase in the IL-17 groups when compared to the untreated group (Fig. 4f).

The difference between exAGNx1 overall release (calculated as AUC) from 25 ng/mL and 100 ng/ml IL-17A-treated explants was not statistically significant (Mann-Whitney test, *p* = 0.1782). Same was true for C2M release (Mann-Whitney test, *p* = 0.0519).

### IL-17 induces MMP release from full depth bovine articular cartilage in our explant system

**Fig. 5:**
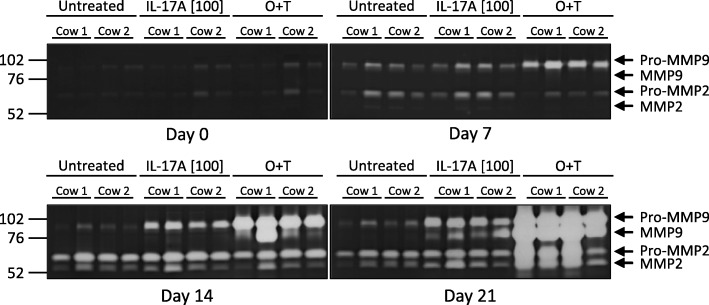
Gelatin zymography assessment of MMP2 and MMP9 release from bovine articular cartilage explants. Conditioned media from two explants for each condition from two separate animals were analyzed at days 0, 7, 14 and 21. Pro-MMP9 (~ 92 kDa), active MMP9 (~ 82 kDa), pro-MMP2 (~ 72 kDa), cleaved MMP2 (~ 63 kDa) are indicated with arrows

To further confirm our biomarker results, conditioned media from days 0, 7, 14 and 21 were harvested and gelatinase release and activity were assessed by zymography (Fig. 5). Pro-MMP9 was detected in all samples at day 7, with higher levels in O + T treated samples. At day 14, pro-MMP9 levels increased in the cytokine-treated groups but not in the control group. Additionally, the active MMP9 form was detected in O + T treatment group at day 14, and in both O + T and IL-17A[100 ng/ml] groups at day 21, but not at day 14 or day 21 of the untreated group. Pro-MMP2 was detected in all samples at all timepoints except day 0, with increased levels in the O + T treatment group at day 21. The active MMP2 form was detected at day 14 and day 21 in all treatment groups, with the bands appearing more intense in the cytokine-treated groups at day 21.

### Proteomics identifies differentially expressed proteins upon IL-17 treatment

**Fig. 6:**
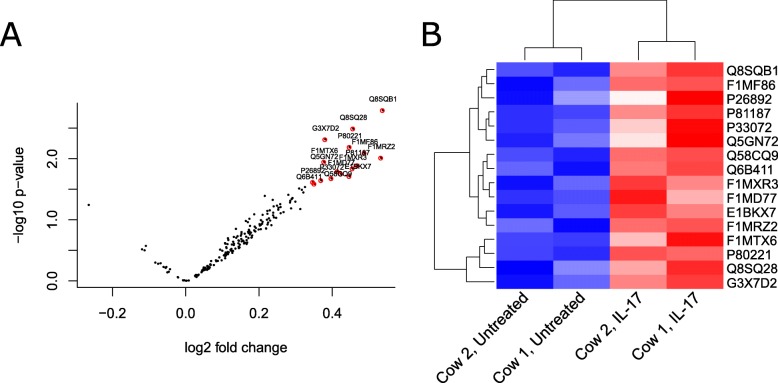
Differential expression analysis of proteins released into BEX conditioned media. Conditioned media from days 10, 14, 19 and 21 was analyzed and relatively quantified by mass spectrometry. AUC was calculated as an indication of total signal, and untreated and IL-17A treatment group AUC values were compared using ROTS statistical test with FDR < 0.05 considered significant. Only proteins with relative abundance values in both treatment groups were considered. **a** a volcano plot showing the relationship between fold changes and *p*-values, and **b** a heatmap with hierarchical clustering showing the expression levels of the 16 differentially expressed proteins as colors

**Fig. 7:**
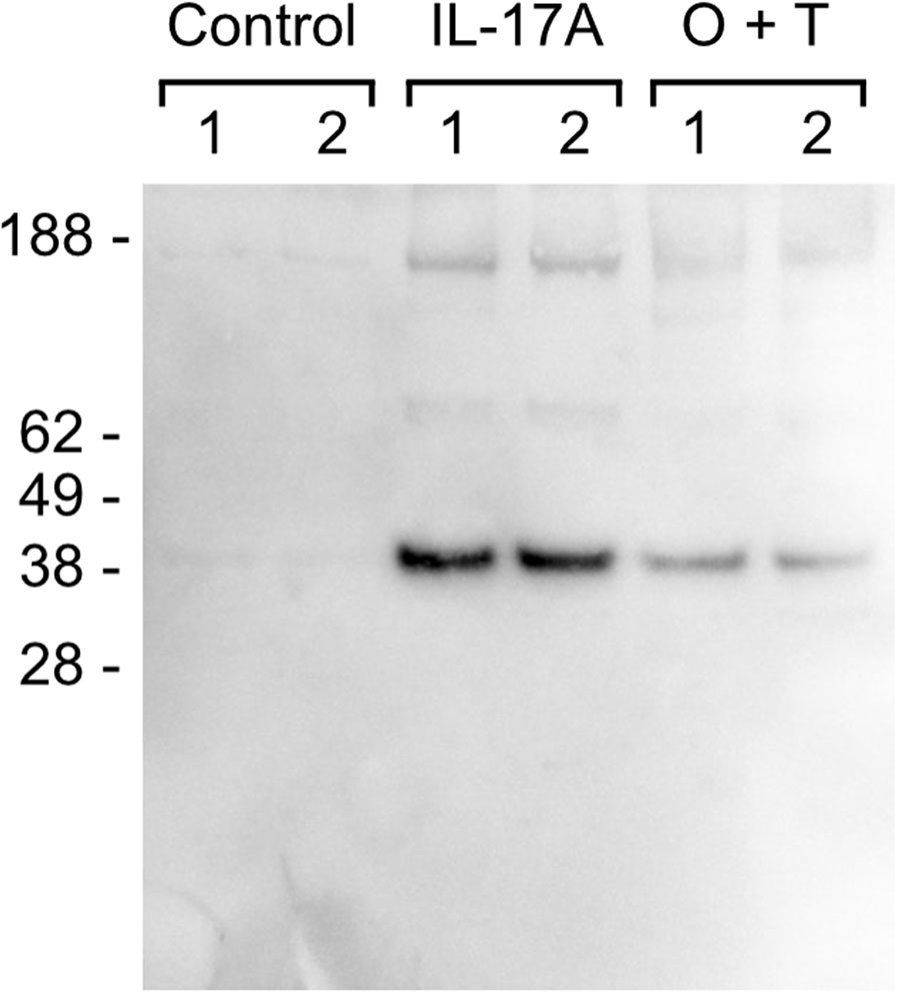
CHI3L1 release into BEX conditioned media in response to cytokine treatment in explants from two independent animals. Conditioned media from control or cytokine-treated (IL-17A [100 ng/ml] or OSM [10 ng/ml] + TNFα [20 ng/ml]) explants, from days 10, 14, 19 and 21 was pooled and the same volume of pooled media (20 μl) was analyzed by Western blotting. Numbers 1 and 2 in the figure denote the two animals. An intense band, that corresponds to CHI3L1 (MW: 39 kDa), was observed in the 100 ng/ml of IL-17A as well as the O + T groups

To obtain an unbiased global protein release profile from the IL-17A-treated articular cartilage we turned to proteomics. In brief, conditioned media from days 10, 14, 19 and 21 from two animals (two explants per each timepoint of each treatment group) was ethanol-precipitated to remove phenol red, trypsin-digested and analyzed by shotgun mass spectrometry. After protein identification and label-free quantification based on the top 3 most intense peptides for the protein group, the data was filtered so that only proteins present in all treatments, all timepoints, in both animals remained to obtain a data matrix with no missing values. This resulted in rejection of 175 proteins, and a final dataset of 199 proteins (Supplementary data) on which the differential expression analysis between untreated and IL-17A[100 ng/ml] groups could be performed using ROTS statistics. 16 proteins were differentially expressed in the IL-17A vs untreated group with an FDR < 0.05 (Fig. 6a-b, Table 1). The upregulation of CHI3L1 in IL-17- and O + T- treated explant conditioned media was confirmed by Western blotting (Fig. 7).

**Table 1:** Proteins upregulated in the conditioned media of IL-17A [100 ng/ml] samples when compared to untreated (adjusted *p*-value < 0.05)

Accession​	Description​	Gene	ROTS-statistic​	*P*-value​	FDR​
Q8SQB1​	C-C motif chemokine 20	CCL20	− 75.2501	0.0016	0​
Q8SQ28​	Serum amyloid A protein	SAA3	− 75.1999	0.0032	0​
G3X7D2​	Chitinase-3-like protein 1	CHI3L1	− 70.1621	0.0048	0​
P80221​	C-X-C motif chemokine 6	CXCL6	− 69.4439	0.0064	0​
F1MF86​	Latent-transforming growth factor beta-binding protein 2	LTBP2	− 68.0478	0.0080	0​
F1MRZ2​	A disintegrin and metalloproteinase with thrombospondin motifs 5	ADAMTS5	− 63.8272	0.0096	0​
F1MTX6​	Matrix metalloproteinase	MMP3	− 61.1325	0.0112	0​
P81187​	Complement factor B	CFB	− 60.5126	0.0128	0​
F1MXR3​	ADAMTS like 4	ADAMTSL4	− 59.5466	0.0144	0​
Q5GN72​	Alpha-1-acid glycoprotein	AGP	− 56.2470	0.0160	0​
F1MD77​	Laminin subunit gamma 1	LAMC1	−54.4637	0.0176	0​
E1BKX7​	Filamin B	FLNB	−54.1942	0.0191	0​
P33072​	Protein-lysine 6-oxidase	LOX	−50.8668	0.0207	0​
Q58CQ9​	Pantetheinase	VNN1	−50.7081	0.0223	0​
P26892​	Interleukin-6	IL6	−50.1170	0.0239	0​
Q6B411​	Lysozyme C, milk isozyme	LYZ	−45.5667	0.0255	0​

## Discussion

We have shown that IL-17A can induce articular cartilage degradation in our bovine stifle joint full-depth cartilage explant model, as measured by proteoglycan degradation and cartilage turnover biomarker release. A remarkable GAG content reduction was observed in tissue at both lower and higher doses of IL-17A and there was a corresponding increase in GAG content in the conditioned media. This is in agreement with previous, shorter (up to 14 days) explant model studies, where stimulation of bovine nasal cartilage, or cartilage from bovine and porcine metacarpophalangeal or human knee joints with IL-17 led to a dose-response increase in nitric oxide production, increase in GAG release from tissue, and a decrease in matrix synthesis [39, 40, 51, 52]. It was previously reported that IL-17 effects on GAG turnover were attenuated by aggrecanase inhibitor actinonin and that exposure of bovine articular cartilage explants to IL-17 upregulated ARGSV aggrecan fragment release [38]. We confirm this finding by measuring an elevated NITEGE aggrecan fragment release from IL-17A-treated explants, which is the C-terminal neo-epitope of ADAMTS-mediated RNITEGE^373^ ↓^374^ARGSVIL cleavage in the G1 domain of aggrecan [45]. We also find elevated ADAMTS5 levels in IL-17A explant media as compared to untreated in the mass spectrometry analysis (accession F1MRZ2). In contrast to the catabolic control (O + T), IL-17 appears to induce a milder, but prolonged increase in the NITEGE degradation fragment release that continues above untreated levels at day 21 of culture.

In addition to aggrecan degradation, IL-17 stimulation of bovine nasal cartilage explant cultures previously had induced a type II collagen release into conditioned media, measured by a hydroxyproline assay at day 7 and day 14 of a 14-day culture, and upregulated the mRNA of MMP1, MMP3 and MMP13 [39]. MMP3 was increased in our IL-17A explant conditioned media, confirming the upregulation of MMP3 at a protein level. We also see an increase in MMP9 release, which has not been previously shown in articular cartilage explants. Furthermore, we measure an increase in MMP-mediated type II collagen fragment (C2M) release in our IL-17A-treated explant conditioned media, definitively showing that at least some of the type II collagen release is MMP-mediated. The IL-17 induced type II collagen degradation occurs later than by O + T, thus in the future it may be worth extending the culture period to 4 weeks, considering a previous report that bovine explants were less responsive to IL-17 compared to porcine in terms of matrix breakdown [40] as well as our delayed C2M responses (compared to O + T). One reason could be the extended exAGNx1 observed, since it is suggested that aggrecan plays a protective role in preventing degradation of collagen fibrils [53]. In addition to MMP-mediated type II collagen fragment release we also show an increase in MMP9 release (by zymography) in our IL-17-treated explant conditioned media. The MMP9-mediated type III collagen degradation fragment C3M release showed some induction in IL-17 [100 ng/ml] explant group at the very last days of culture.

Previously, it has been shown by Western blotting and ELISA assays that in normal human chondrocytes IL-6 was upregulated in response to IL-17 through at least in part activation of MAP kinases and NFkB, and this upregulation was inhibited by dexamethasone [51, 54]. We confirm this in our explant model and additionally find upregulation of CXCL6, CCL20 chemokines in IL-17A-treated explant conditioned media. CXCL6 and CCL20 gene expression has been previously reported significantly increased in IL-1β-treated human chondrocytes and CCL20 has also been reported to be abundantly expressed in OA cartilage sections [55, 56]. CCL20 mRNA was shown to increase after chondrocyte (from healthy cartilage) stimulation with IL-6 or IL-17 and the treatment of normal, healthy cartilage with CCL20 lead to an elevated proteoglycan release from the tissue [56].

Other immune response mediators – complement factor B and SAA were also upregulated in IL-17A-treated explant conditioned media. Complement has a role in OA and factor B synthesis has been previously shown to be regulated by IL-17 in fibroblasts [57–60]. Struglics et al. (2016) have shown that synovial fluid from OA joints contains substantial amounts of complement factors in comparison with the reference group (60). In OA cartilage, the release of ECM components and motifs (e.g. degradation products of type II collagen or other proteins) into the synovial fluid – the so-called damage-associated molecular patterns (DAMPs) – result from the dysregulated activity of various proteases; many of these pathways show interactions with complement factors [58, 59]. Acute-phase SAA levels are elevated in OA and RA patient plasma and synovial fluid, and in OA increased SAA levels correlate positively with the Kellgren & Lawrence grade [61].

CHI3L1 (YKL-40), which was upregulated in the conditioned media of our IL-17A explant group, is also involved in joint pathology – its elevation in synovial fluid independently and positively related to WOMAC pain (r = 0.531, *p* = 0.001), physical disability (r = 0.380, *p* = 0.025), and total scores (r = 0.407, *p* = 0.01) in knee OA patients [62]. CHI3L1 has also been previously shown to be released from OA cartilage, and its levels in the synovial fluid of OA patients have been reported to correlate positively with MMP1, MMP3, IL-6 and IL-17 [63]. Additionally, its release from articular chondrocytes has been previously induced by other pro-inflammatory cytokines (e.g. IL-1, TNFα) and LPS, in the latter case through TLR4 and NFkB [64, 65]. TLR4 activation is required for IL-17-induced multiple tissue inflammation in C57BL/6 mice, and TLR4 deficient DBA1J mice show decreased IL-17 levels and incidence of collagen-induced arthritis [66, 67].

### Study limitations

We have chosen to work with bovine tissue due to the limited availability of healthy human tissue and have focused on stifle joint/knee articular cartilage, as this is the joint most commonly affected by OA. However, the bovine genome is not as well characterized as the human genome. For example, the primers for bovine IL-17 receptors were designed from predicted mRNA sequences, as verified sequences not yet exist. Ideally, the receptor expression should be further confirmed by histology or western blotting, however, no verified bovine IL-17 receptor specific antibodies are available. Additionally, there is limited availability of suitable antibodies to confirm proteomics results.

We have used two IL-17 two concentrations, as it has been reported by Cai et al. (2001) that higher concentrations of IL-17 were required to elicit a NO and matrix breakdown response in bovine compared to porcine explants. In the future, it could be worth trying co-stimulation with other pro-inflammatory cytokines as, previously in bovine explants, IL-17 combination with TNFα resulted in synergy and, with LIF – in additive effect, respectively [39, 68].

The relatively low number of proteins (*n* = 16) identified as statistically significant is in part due to the low power of this pilot study, and in part due to the strict filtering criteria, which omitted proteins with missing LFQ values. In the future, use of a Bayesian selection model could be tried out to reduce such data loss [69]. Another major limitation of the study is that like the bovine genome, the bovine proteome is still relatively poorly characterized. UP000009136 contains multiple sequences per protein of varying annotation quality, with generally not that many reviewed proteins in SwissProt (*n* = 6006) when compared to human (*n* = 20,402).

## Conclusions

To summarize, IL-17A can induce articular cartilage degradation in our bovine full-depth explant model. Compared to previous studies, our model brings value in the extended tissue culture - previously in literature the duration of the IL-17 ex-vivo models were limited to 14 days. We also take more frequent measurements (every 2–3 days instead of every 7 days of the 14-day study), which gives a better overview of the undergoing processes. The exAGNx1 biomarker, and potentially also C2M if the culture period would be extended to four weeks, appear to be good tools for assessing cartilage damage in this model. Finally, the confirmation of previously published findings supports the validity of the model as a tool for IL-17 mediated cartilage catabolism exploration by mass spectrometry, and for cartilage degradation biomarker discovery. The model will allow us to further study protein cleavages and to time-profile the IL-17-induced articular cartilage degradome, as well as to look for neo-epitopes specific for IL-17-mediated articular cartilage degradation.

## Supplementary information

**Additional file 1: Figure S1.** Dose-response test of bovine articular cartilage explants (BEX) by assessing MMP-mediated type II collagen degradation on day 14 of bovine explant culture. Data from 1 cow, 6 individually cultured explants per each treatment. ULMR denotes the upper measurement limit of the assay.

**Additional file 2: Table S1.** 199 proteins selected for differential expression analysis sorted by PEP score. **Table S2.** Mean log2 abundances of 199 proteins selected for differential expression analysis.

## Data Availability

The raw data used and/or analyzed during the current study could not be deposited in a public repository, but are available from the corresponding author or senior authors on request after establishment of a research agreement.

## Abbreviations

ADAMTS: A Disintegrin And Metalloproteinase with Thrombospondin Motifs
BEX: Full-Depth Bovine Articular Cartilage Explants
COX-2: Cyclooxygenase 2
DAMPS: Damage-Associated Molecular Patterns
DMMB: Dimethyl Methylene Blue
DMOAD: Disease-Modifying Osteoarthritis Drug
ECM: Extracellular Matrix
ELISA: Enzyme-Linked Immunosorbent Assay
EMA: European Medical Agency
FDA: Federal Drug Administration
GAG: Glycosaminoglycan
IL-17: Interleukin-17
IL-17R: Interleukin-17 Receptor
IL-6: Interleukin-6
LLMR: Lower Limit of Measurement Range
MAPK: Mitogen-Activated Protein Kinase
MMP: Matrix Metalloproteinase
NFΚB: Nuclear Factor Kappa Light Chain Enhancer of Activated B Cells
NO: Nitric Oxide
OA: Osteoarthritis
OSM: Oncostatin M
PGE_2_: Prostaglandins
RA: Rheumatoid Arthritis
TBST: Tris-buffered saline, 0.1% Tween 20
TMB: 3,3',5,5'-Tetramethylbenzidine
TNFα: Tumor Necrosis Factor Alpha
ULMR: Upper Limit of Measurement Range
