# Supplementary figures and images for: Characterization of the interleukin-17 effect on articular cartilage in a translational model: an explorative study
Source: BMC Rheumatol. 2020 May 12;4:30. doi: 10.1186/s41927-020-00122-x (PMC7216541; doi:10.1186/s41927-020-00122-x)

Day 14

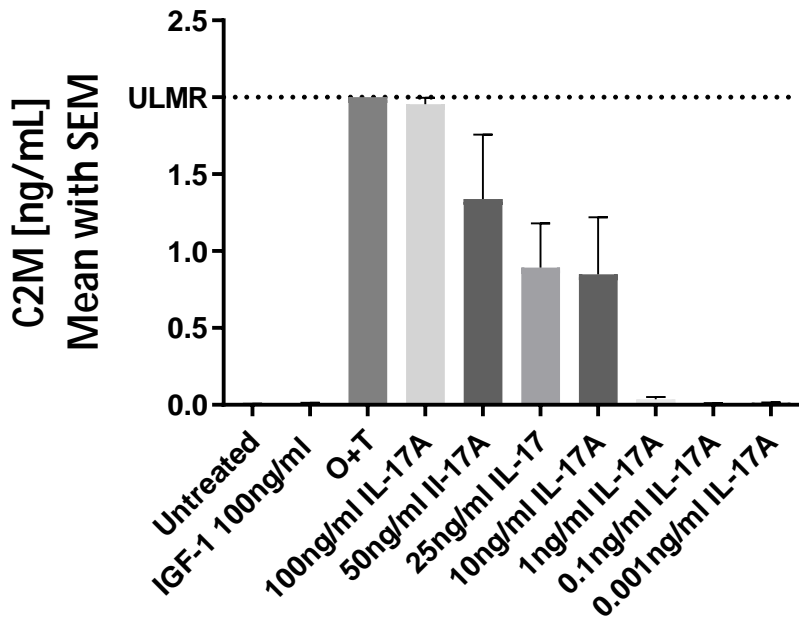

Supplement: Supplementary file 1 — Additional file 1: Figure S1. Dose-response test of bovine articular cartilage explants (BEX) by assessing MMP-mediated type II collagen degradation on day 14 of bovine explant culture. Data from 1 cow, 6 individually cultured explants per each treatment. ULMR denotes the upper measurement limit of the assay. [file 41927_2020_122_MOESM1_ESM.pdf]
